# Is It Time for a New Algorithm for the Pharmacotherapy of Steroid-Induced Diabetes?

**DOI:** 10.3390/jcm13195801

**Published:** 2024-09-28

**Authors:** Aleksandra Ostrowska-Czyżewska, Wojciech Zgliczyński, Lucyna Bednarek-Papierska, Beata Mrozikiewicz-Rakowska

**Affiliations:** Department of Endocrinology, Centre of Postgraduate Medical Education, Marymoncka St. 99/103, 01-813 Warsaw, Poland

**Keywords:** diabetes, glucagon-like peptide 1 agonists, glucocorticoids, pharmacotherapy algorithm, steroid-induced diabetes

## Abstract

Glucocorticoids (GS) are widely used in multiple medical indications due to their anti-inflammatory, immunosuppressive, and antiproliferative effects. Despite their effectiveness in treating respiratory, skin, joint, renal, and neoplastic diseases, they dysregulate glucose metabolism, leading to steroid-induced diabetes (SID) or a significant increase of glycemia in people with previously diagnosed diabetes. The risk of adverse event development depends on the prior therapy, the duration of the treatment, the form of the drug, and individual factors, i.e., BMI, genetics, and age. Unfortunately, SID and steroid-induced hyperglycemia (SIH) are often overlooked, because the fasting blood glucose level, which is the most commonly used diagnostic test, is insufficient for excluding both conditions. The appropriate control of post-steroid hyperglycemia remains a major challenge in everyday clinical practice. Recently, the most frequently used antidiabetic strategies have been insulin therapy with isophane insulin or multiple injections in the basal–bolus regimen. Alternatively, in patients with lower glycemia, sulphonylureas or glinides were used. Taking into account the pathogenesis of post-steroid-induced hyperglycemia, the initiation of therapy with glucagon-like peptide 1 (GLP-1) analogs and dipeptidyl peptidase 4 (DPP-4) inhibitors should be considered. In this article, we present a universal practical diagnostic algorithm of SID/SIH in patients requiring steroids, in both acute and chronic conditions, and we present a new pharmacotherapy algorithm taking into account the use of all currently available antidiabetic drugs.

## 1. Introduction

Glucocorticoids (GS) are drugs widely used in the treatment of respiratory diseases, skin lesions, rheumatoid arthritis, and renal diseases. Interestingly, it is estimated that as many as 1–1.8% of all patients are treated with GS [1]. Among hospitalized patients, this number rises to 10% [2]. The therapy usually lasts less than 5 days; however, 22% of patients use GS for longer than 6 months [3]. Despite their positive impact on disease control, prolonged treatment with GS is associated with side effects such as osteoporosis, sarcopenia, hypertension, cataracts, glaucoma, weight gain, hyperglycemia, and steroid-induced diabetes (SID). It is estimated that glucose metabolism disorders appear in 1.5–47% of patients treated with GS [4]. However, the scale of the problem might be underestimated, since the most commonly used screening test is the fasting blood glucose level, which is not sufficient for the exclusion of steroid-induced hyperglycemia and diabetes [5]. Typically, during GS treatment, an increased postprandial level coexists with a normal or slightly increased fasting glucose level. The impact on postprandial glucose level depends on the strength and time of GS action [6]. Therefore, it is necessary to adapt the pharmacokinetic profile of antidiabetic drugs to the dynamically changing glycemic profile.

## 2. Pathogenesis of GS-Induced Glucose Metabolism Impairment

GS work in a fast-acting mechanism through attachment to the membranes of immune cells, inducing an extra-genomic anti-inflammatory effect [7]. However, after a few hours, they affect the genome, leading to changes in metabolism and a reduction in inflammation [8].

There is no information about the maximal duration of this effect; however, taking into account those patients in whom hyperglycemia is transient after the discontinuation of GS therapy, increased glucose levels in most cases may last up to 3 months.

The genomic mechanism of GS prevails during treatment with low (<7.5 mg prednisone) and intermediate doses (7.5–30 mg prednisone) [9]. Non-genomic mechanisms activate during the application of prednisone dosages above 100 mg and reach the greatest effects around 250 to 500 mg [10].

There are several mechanisms involved in the development of steroid-induced hyperglycemia (SIH) and steroid-induced diabetes. Potential mechanisms are listed below:

(a) GS have an antagonistic reaction to insulin. They stimulate hepatic glucose production and lipolysis in adipose tissue, which leads to increased insulin resistance and impairment of insulin secretion from ß -cells in the pancreas [11,12]. In addition, they intensify the diabetogenic impact of glucagon, growth hormone, and catecholamines [4].
(b) GS induce lipid redistribution from peripheral to abdominal fat. They also stimulate pre-adipocytes differentiation to adipocytes, triglyceride synthesis, and adipocytes hypertrophy [13].
(c) GS have an impact on the incretin effect [4]. In the animal model, treatment with GS led to reduced mRNA stability of the preproglucagon gene, the precursor of glucagon-like peptide-1 (GLP-1), decreasing its concentration [14]. The results in humans are ambiguous; however, it is postulated that treatment with GS weakens postprandial GLP-1-dependent insulin release [4].
(d) GS can impair pancreatic cell function. They reduce insulin secretion from β-cells due to the restriction of uptake and the oxidation of some substrates such as glucose, and the suppression of cell membrane depolarization [5]. In addition, they promote β-cell apoptosis [15]. Moreover, GS increase glucagon secretion from alfa cells, which leads to hyperglycemia [4].
(e) GS increase the mRNA expression of neuropeptide Y (NPY) and agouti-related peptide (AgRP) neurons in the arcuate nucleus of the hypothalamus, which leads to increased appetite in rodents [16]. It is postulated that the increased intake of comfort food (induced by GS) through weight gain intensifies insulin resistance.
(f) GS impact on sodium-glucose co-transporter-2 (SGLT2) is uncertain, due to a lack of studies on the treatment with SGLT2 inhibitors as monotherapy [17].
(g) GS impair insulin-mediated capillary recruitment, which may contribute to the adverse effects of steroids on glucose metabolism. An impairment of capillary recruitment is analogous to insulin resistance, increased resistin level, and increased postprandial glucose level [18].

**Figure 1:**
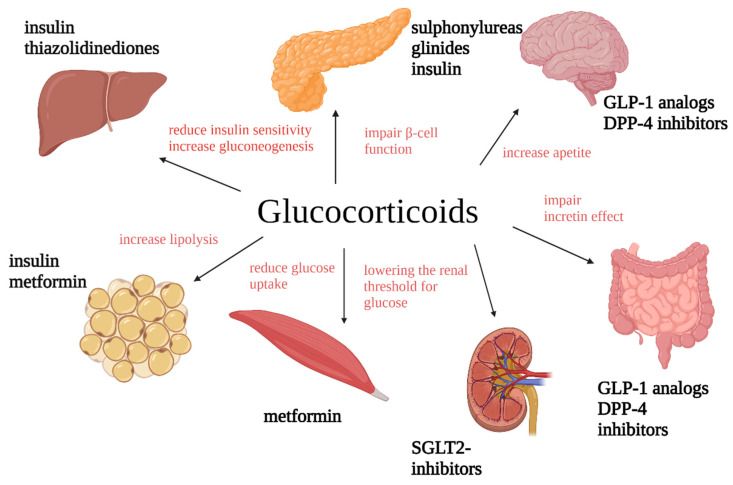
Mechanisms of GS impact on glucose metabolism and the potential pharmacological target points to reduce glycemia. GLP-1: glucagon-like peptide-1; DPP-4: inhibitors-dipeptidyl peptidase 4 inhibitors; SGLT2i: sodium glucose cotransporter-2 inhibitors.

Figure 1 presents the mechanisms of GS impact on glucose metabolism and the potential pharmacological target points to reduce glycemia.

Despite such diverse mechanisms impairing glucose tolerance during GS treatment, not all patients develop SID or SIH. Their incidence is estimated to vary widely: between 10 and 50% [19]. When starting GS therapy, attention should be given to risk factors of glucose metabolism disruption, which increase the probability of developing diabetes even without GS. Table 1 presents the risk factors that a physician should consider when treating a patient undergoing therapy with glucocorticoids that may lead to the earlier onset of diabetes.

## 3. Pharmacokinetic and Pharmacodynamic Properties of Glucocorticoids as the Basis for the Optimal Selection of Antidiabetic Drugs

Short-acting GS include hydrocortisone (HC) (duration of action 8–12 h), intermediate-acting GS are comprised of prednisolone (duration of action 12–36 h) and methylprednisolone (half-life of 18–40 h) which are 4–5 times more potent than HC, and finally long-acting GS such as dexamethasone and betamethasone are 25 times more potent than HC (duration of action 36–54 h) [3,4,5,6,7,8,9,10,11,12,13,14,15,16,17,18,19,20]. According to Polish Diabetes Society guidelines, doses of GS as big as 20 mg of hydrocortisone equivalent have no significant impact on glucose level control [36]. However, it is increasingly being pointed out that there is no safe dose of glucocorticoids, and even administration of glucocorticoids in a dose equivalent to 5 mg of prednisone may cause diabetes [37]. It seems that the final metabolic effects depend on the duration of the treatment and individual predispositions.

To choose the most suitable GS, it is necessary to take into account their pharmaco-kinetic and pharmacodynamic properties. Table 2 contains the characteristics of various GS.

The final glycemic profile observed in a patient is the result of its potential half-life in the bloodstream and its potency, as well as the frequency of its administration. An example could be the administration of hydrocortisone, which theoretically should cause one glycemic peak, but in acute conditions, e.g., in the case of bronchial tree obstruction, it is usually administered at least 2–3 times a day. Therefore, the increase in glycemia during administration of this agent at such a rhythm persists throughout the day.

It should be emphasized that in some diseases, steroids are administered in the form of pulses, i.e., large doses of steroids. It has been proven that this form of glucocorticoid administration is usually associated with higher effectiveness in maintaining low clinical disease activity than the constant administration of continuous doses [5,6,7,8,9,10,11,12,13,14,15,16,17,18,19,20,21,22,23,24,25,26,27,28,29,30,31,32,33,34,35,36,37,38,39]. In most patients, it is necessary to repeat the steroid pulse several times. Each subsequent pulse causes the glycemic threshold to be adjusted to a higher level, which ultimately results in the need to modify antidiabetic therapy. Table 3 shows examples of glucocorticoid dosing in some clinical situations.

**Figure 2:**
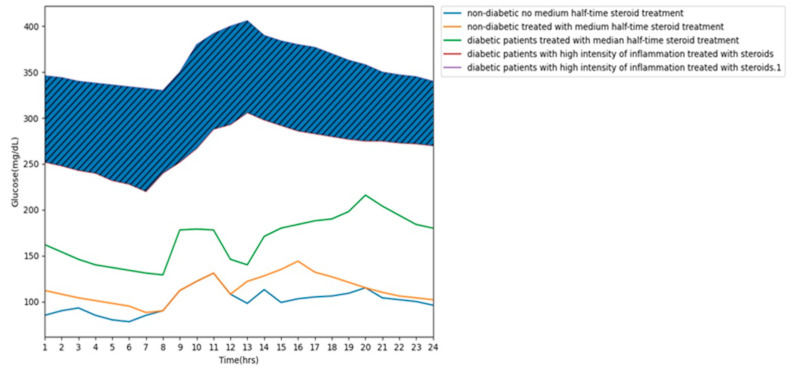
Glycemic patterns during therapy with intermediate-acting glucocorticoids based on [38] and authors’ own experiences.

In patients treated with GS, a characteristic glycemic profile is observed in which fasting glycemia is usually only slightly increased, and an increase in glycemia predominantly appears at noon and in the afternoon. In the evening, a decrease in serum glucose is observed. Even though exacerbation of the basic process which is associated with inflammation is so high that a significant increase in both pre- and postprandial glycemia is observed in the daily profile, a disproportion between morning and afternoon glycemia can still be observed. The above dependencies are illustrated in Figure 2.

## 4. The Influence of Different Pharmaceutical Forms on Glucose Metabolism

GS may be administered in numerous ways. However, oral and intravenous administration are most commonly used in clinical practice.

(a) Oral and intravenous GS

Some studies point out that this form of therapy is associated with up to 2% of diabetes cases, but these studies comprise the diagnosis of diabetes based on fasting blood glucose level [48]. However, while taking into account diagnostic procedures for postprandial hyperglycemia, it is estimated that treatment with oral GS is associated with an increased risk of diabetes by up to 36% [49]. It is estimated that 25% of patients treated with high doses of prednisolone (above 30 mg/dL) develop SIH or SID [50]. Moreover, in the case of implementation of high doses of prednisone (1 mg/kg/d) for 8 weeks, 40.6% of patients developed diabetes. Interestingly, most of the diagnoses of SID/SIH were made in the first week (34.3%) and the risk did not increase after 12 weeks [39].

In patients treated with low-dose steroids, the risk of developing glucose metabolism disorders is ambiguous [1]. Panthakalam et al. reported that in patients with rheumatoid arthritis treated with a low dose of prednisone (mostly 7.5 mg), the incidence of SID was 8.8% [51]. On the other hand, among the participants of four randomized controlled trials treated with 5–10 mg of prednisone for 2 years, nobody developed SID [52,53,54,55].

In the UK primary care database (The Clinical Practice Research Datalink), using an equivalent dose of 5 mg of prednisolone in the treatment of rheumatoid arthritis for 1, 3, and 6 months was significantly associated with the development of steroid-induced diabetes hazard ratios of 1.20, 1.43, and 1.48, respectively, compared to a control group [56].

An intake of even a single dose of GS has an impact on glucose level control. It is usually associated with increasing postprandial glucose levels without impacting the fasting glucose level [1,2,3,4,5]. It is interesting to note that the probability of developing SID or SIH depends on the duration of the treatment, irrespective of prior history of diabetes mellitus [49,50,51,52,53,54,55,56,57]. Among patients treated with 30 mg prednisone daily for only 7 days, a reduction in insulin sensitivity was observed up to 60% [58]. However, Movahedi et al. described the risk of development of diabetes in patients treated with a dose equivalent to 5 mg of prednisone only for treatment lasting up to 6 months [56].

As a rule, the incretin effect does not differ among patients receiving low or moderate daily doses of glucocorticoids orally or intravenously. Differences in the incretin effect may be observed with pulsatile administration of glucocorticoids. For glucocorticoid administration in aerosol, nasal, or topical forms, the final incretin effect is most influenced by the bioavailability of the preparation.

(b) Intraarticular GS

Data on the prevalence of SID after the administration of intraarticular GS are limited. Most of the described cases of glucose metabolism impairment during intraarticular GS treatment are related to SIH [59,60,61]. A possible reason is the short duration of treatment. Waterbook et al. described a transient increase of blood glucose levels as high as 518 mg/dL, occurring within 1 to 5 days after GS administration [59]. The most common blood glucose levels ranged between 125 and 320 mg/dL and normalized in up to 10 days [59]. Interestingly, higher blood glucose levels after GS injection were observed in patients with type 1 diabetes mellitus (T1DM) and other types of diabetes treated with insulin [62,63]. In a study by Akin-Takmaz et al., groups of diabetic and non-diabetic patients treated for at least 3 months with the intraarticular injection of triamcinolone into the glenohumeral joint due to adhesive capsulitis and shoulder pain were compared [64]. A higher increment of the fasting blood glucose level on day 1 after GS injection in diabetic patients versus the non-DM group was observed, and longer blood glucose elevations were observed [64].

(c) Inhaled GS

Most of the newest studies show that treatment with inhaled glucocorticoids (ICS) does not increase the risk of developing SIH or SID independently of therapy duration and GS dose [65,66,67,68,69,70]. However, some researchers found an association between GS and glucose metabolism impairment. Using ICS over 1 year is associated with an increased risk of SID [71]. Among people regularly treated with ICS due to chronic obstructive pulmonary disease for at least 6 months with a high dose (640 micrograms of budesonide equivalent daily), the median time needed to develop SID was 1.3 years (0.3–6.7 *p* < 0.0088), whereas in those treated regularly with low doses, the median was 2.1 years (0.3–8.2 *p* < 0.001) [72]. Price et al. stated that during high doses of ICS therapy (500 µg/day or greater fluticasone propionate equivalent), there is a statistically significant risk of SID development, progression of diabetes mellitus, and the necessity of modifying diabetes treatment with an insulin introduction [73].

(d) Intranasal GS

The systemic bioavailability of intranasal glucocorticoids (INS) is associated with the risk of SIH. In the case of treatment with INS with low systemic bioavailability (1%), such as mometasone furoate and fluticasone propionate, no statistically significant risk of SIH during therapy for at least 3 months was observed. In a patient treated with triamcinolone acetonide (bioavailability 46%), a significantly greater increase in fasting glucose levels was observed. However, none of the INS had effects on HbA1c and glucose levels in diabetic patients [74].

(e) Topical GS

Topical glucocorticoids (TCS) are widely used in dermatology. Two case–control studies performed in patients treated with TCS revealed an increased risk of SID. Moreover, significant dose–response and potency–response relationships between the risk of developing SID during TCS therapy were observed [75].

Data regarding the incretin effect following the intake of various forms of glucocorticoids are limited. In 11 healthy participants, a statistically significant reduction in the incretin effect from 70% to 40% was observed after 12 days of orally administered 37.5 mg prednisolone once daily [76]. In a study by Jensen et al., patients receiving intravenous dexamethasone 2 mg twice daily for 5 days or more exhibited an impaired incretin effect, with percentages decreasing from 71 ± 3.2% and 67 ± 4.6% to 58 ± 5.2% and 32 ± 8.8%, respectively (*p* < 0.05 between the two groups) [77]. An increase in pancreatic alpha-cell dysfunction was also observed in both groups, which may signal an early risk of developing diabetes [77].

## 5. Should Glycemic Targets for Patients Treated with GS Be More Liberal? How Does This Translate to the Glycemia Monitoring Process?

According to American Association of Clinical Endocrinology (AACE) guidelines, glycemic targets for individuals on glucocorticoid therapy in domiciliary care and wards should be less than 140 mg/dL pre-meal and less than 180 mg/dL 2 h post-meal [78]. Moreover, if the risk of hypoglycemia does not rise, more rigorous glycemic goals of 110–140 mg/dL, especially in younger patients with newly diagnosed diabetes, should be achieved [6]. Although the criteria for diagnosing steroid-induced diabetes do not differ from the guidelines for diagnosing diabetes, there are no clear guidelines regarding the glycemic threshold at which drug therapy should be initiated. British guidelines recommend initiating treatment if monitored blood glucose is 200 mg/dL or more [26]. However, there is a lack of recommendations on how and when to initiate the therapy in patients with abnormal fasting glycemia, increased HbA1c percentage, or abnormal oral glucose tolerance test (OGTT) results. It is interesting to note that in the guidelines of the British Society of Diabetology, sulfonylurea derivatives are to be initiated when glycemia exceeds 216 mg/dL. It seems that the key to determining optimal pharmacotherapy is to determine the glycemic thresholds achieved during and after therapy and the method of monitoring.

The British Society of Diabetology proposed an algorithm for glucose monitoring de-pending on the patient’s need for hospitalization (Table 4).

**Figure 3:**
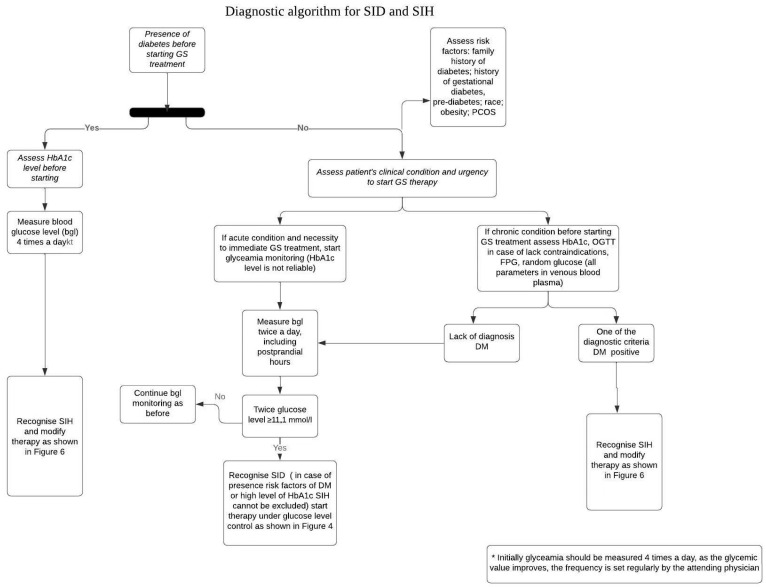
A diagnostic algorithm for SID and SIH based on the present medical history and laboratory markers of diabetes. DM: diabetes mellitus, OGTT: oral glucose tolerance test; FPG: fasting plasma glucose level; GS: glucocorticoids; SID: steroid-induced diabetes; SIH: steroid-induced hyperglycemia; PCOS: polycystic ovarian syndrome.

Until now, a comprehensive diagnostic algorithm that accounts for the potential overlap or coexistence of diabetes resulting from various etiologies has not been presented. Clinicians should be aware of the possibility of Type 1 diabetes induction in patients treated with immunotherapy (e.g., Nivolumab, Durvalumab) for conditions such as melanoma or lung cancer. These patients often receive high doses of GCs in conjunction with biological therapy. Therefore, when administering high doses of GCs, attention should be paid to the risk of ketoacidosis. It is also important to note that if there is a possibility of delaying glucocorticoid therapy, it is advisable to conduct tests to determine the patient’s glycemic status before treatment. Figure 3 presents a diagnostic algorithm for SID and SIH based on the present medical history and laboratory markers of diabetes.

## 6. The Recommendations of Scientific Associations Regarding the Pharmacological Treatment of Hyperglycemia in SIH/SID

According to the Polish Diabetes Society’s guidelines for the treatment of SID, intensive insulin therapy is preferred [36]. However, in the case of correct fasting and preprandial blood glucose levels, rapid-acting human insulins or insulin analogs can be administered. There are no significant differences in blood glucose control between human insulins and insulin analogs [79].

### 6.1. Insulin Therapy

According to American Diabetes Association guidelines, insulin therapy should be initiated when, despite oral drug administration, patients have a persistent glucose level of 180 mg/dL or higher [2]. Taking into account the impact of GS on postprandial glucose levels, short-acting insulin preparations or fast-acting insulin analogs are recommended [80]. There are no significant differences among basal insulins used in treatment—the most common are intermediate-acting neutral protamine Hagedorn (NPH) and long-acting insulin analogs. However, GS with high potency used for a long period tend to increase fasting glycemia as well, and in such clinical situations, intensive insulin therapy is preferred. An NPH insulin is preferred in individuals treated with a once-daily GS regimen. Considering its duration of action and peak serum concentration after 4–10 h of intake, the best glucose control is achieved when administered simultaneously with GS [5]. During treatment with short and intermediate-acting GS, the suggested initial dose of NPH insulin is 0.1 IU/kg and 0.2–0.4 IU/kg, respectively [6,7,8,9,10,11,12,13,14,15,16,17,18,19,20,21,22,23,24,25,26,27,28,29,30,31,32,33,34,35,36,37,38,39,40,41,42,43,44,45,46,47,48,49,50,51,52,53,54,55,56,57,58,59,60,61,62,63,64,65,66,67,68,69,70,71,72,73,74,75,76,77,78,79,80,81]. Interestingly, the results of two randomized studies with the administration of glargine (dose of 0.5 IU/kg or 0.3–0.4 IU/kg) with intermediate-acting GS revealed that there is no difference in glucose control compared to NPH insulin [82,83]. Another proposed scheme comprises the morning administration of basal human insulin with a starting dose of 10 units and an increasing daily dose between 10% and 20% controlled by blood glucose level. However, in some cases, increments of up to 40% are required to achieve therapeutic goals [6].

Insulin therapy should be especially implemented in patients treated with multiple daily doses of GS or long-acting GS [2,84]. In the case of treatment twice a day with intermediate-acting or long-acting GS, the physician options vary from NPH insulin in divided doses to long-acting basal insulin [5]. When treated with long-acting GS and the blood glucose level is above 216 mg/dL in insulin-naive patients NPH insulin should be administered in a dose of 0.3 UI/kg/day, but two-thirds of the total dose should be administered in the morning and the rest of it in the evening [6]. In adults older than 70 years or with impaired renal function (eGFR < 30 mL/min/1.73 m^2^), the insulin dose should be halved and titrated based on glucose level [84].

Independently of daily doses of GS when glucose control targets are not achieved, rapid-acting insulin can be added in a dose of 0.04 IU/kg when preprandial glucose is 200–300 mg/dL or 0.08 IU/kg for preprandial glucose level above 300 mg/dL [6]. It is also possible to switch treatment to premixed insulin with 70% rapid-acting and 30% basal insulin taken simultaneously with GS [6]. Even though premixed insulin is associated with a higher risk of hypoglycemia than the basal–bolus regimen, due to its decreased number of injections, it should be considered in older patients [78]. Even though high doses of GS are utilized in the most severe cases, there is a significant need to use insulin therapy. Oral hypoglycemic agents could normalize hyperglycemia to some extent. An example of such medications are sulfonylureas. The use of insulin in treatment does not exclude the possibility of adding oral medications. If the HbA1c value over 9% persists, even adding an oral agent to the insulin regimen is recommended [80]. The most common combination is the addition of metformin or/and thiazolidinediones, due to the positive effect on reducing insulin resistance [80]. In well-controlled diabetes based on HbA1c level, oral drugs can be used [5].

In patients with pre-existing diabetes mellitus treated with insulin therapy, an incremental increase in insulin dose by up to 20% is recommended [85]. However, especially in patients with type 1 diabetes mellitus, dose adjustment is a dynamic process and insulin might require titration to be increased by 10–20% or the time of administration to be changed from evening to the morning to achieve the therapeutic goals [26]. The rules of choosing insulin therapy are similar to these for already diagnosed steroid diabetes and are based on the number of doses of GS, the hyperglycemic potency of GS, and body weight [1]. According to Shah et al., the insulin dose based on 10, 20, 30, or 40 mg of prednisolone equivalent in the case of treatment with NPH insulin should be 0.1–0.4 IU/kg per day, respectively [3].

### 6.2. Sulphonylureas

The Joint British Diabetes Society recommends treatment initiation with sulphonylureas in patients taking GS once a day if their blood glucose level is above 216 mg/dL [86]. In contrast, Li et al. noted that sulfonylureas are not recommended as a first-line treatment of SID, because of the long-lasting hypoglycemic effects. These drugs are indicated for patients who are treated with intermediate or long-acting GS taken twice a day or more [20,21]. Taking into account the increased blood glucose levels 4–8 h after GS intake, treatment with glinides in individuals taking steroids once a day seems to be a good choice. Glinides reduce postprandial hyperglycemia to some extent, regardless of the dose and duration of GS used [87,88]. However, due to the necessity of frequent dosing and reduced efficacy in insulin resistance, their therapeutic use is limited [5]. It is recommended to add gliclazide 40 mg once a day in the morning and titrate it up to 240 mg until the glycemic target is achieved [26]. In case of glycemia fluctuations above 250 mg/dL, an evening dose of gliclazide can be added. Moreover, the addition of intermediate-acting insulin or drugs lowering insulin resistance such as metformin or thiazolidinediones should be considered, since they can improve glucose level control [26].

### 6.3. SGLT-2 Inhibitors

The growing popularity of flozins and incretin mimetics has to be underlined as a possible pharmacological choice in the therapy of SID and SIH. Initially, their importance in the treatment of SID or SIH was overlooked due to high costs, unknown side effects, and limited availability. The beneficial effects of flozins in terms of reducing cardiovascular risk would be much needed in the clinical setting of GS-treated patients. However, there is limited data on their efficacy in the treatment of steroid-induced hyperglycemia and diabetes, because they have never been tested in this indication alone [5]. When adding dapagliflozin to hypoglycemic therapy during steroid treatment, minimal improvement in glucose metabolism was observed [89].

### 6.4. Incretin Mimetics

Citing research indicating a reduction in the incretin effect in patients undergoing glucocorticoid therapy even after just five days of treatment, in an era of the widespread availability of incretin mimetics, their application should always be considered in the event of post-steroidal glycemic disturbances [76,77]. It has been demonstrated that GLP-1 receptor agonists (GLP-1RAs) enhance peripheral insulin sensitivity, increase insulin secretion from β-cells, stimulate β-cell proliferation, and reduce their apoptosis [90,91,92]. By inhibiting glucagon release from pancreatic alpha cells, incretin mimetics also counteract corticosteroid-induced glycogenolysis [93,94].

It has been observed that the analog GLP-1 receptor exenatide improves steroid-induced postprandial hyperglycemia in patients with SIH and SID [95,96]. Moreover, dulaglutide can help reduce the insulin dose and number of injections in patients treated with the basal–bolus scheme [97]. However, they have a higher likelihood of inducing hypoglycemia than DPP-4 inhibitors in patients with previously diagnosed diabetes mellitus [98].

Due to a half-life of 4–6 h and positive impact on cardiovascular risk, GLP-1 receptor analogs should be considered especially in people treated with one-dose intermediate-acting GS [5]. Similar observations were made about the treatment of SID with DPP-4 inhibitors, but due to their lower efficacy, the final hypoglycemic effect is much less pronounced. DPP-4 inhibitors lead to higher improvements in glucose level control compared with metformin [78]. It has been observed that DPP-4 inhibitors have greater efficacy in the treatment of SID versus SIH; however, their effectiveness in the treatment of SIH is debatable [5]. Tajiri et al. described worsening control of diabetes and relapses in patients treated with sitagliptin after 6 months [99].

The additional beneficial effect of GLP-1 agonists and DPP-4 inhibitors is the decrease of glucose levels after lunch and supper without the risk of hypoglycemia [78]. Taking into account the action profile of incretin mimetics and their impact on reducing insulin resistance, their combination with insulin therapy could allow for a reduction in the insulin dose [4,98]. Interestingly, a study by Ritzel et al. shows that treatment with GLP-1 ana-logs in patients with diabetes associated with Cushing syndrome has a similar impact on glucose as in patients with type 2 diabetes [100]. Taking into account the impact of GS on the reduction concentration of GLP-1 in rats, the use of GLP-1 analogs in steroid-induced diabetes should be a good choice [14]. Considering the physiological changes that occur after glucocorticoid administration—impaired secretory function of pancreatic beta cells, decreased sensitivity of pancreatic cells to incretins, reduced intestinal synthesis of incretins, and increased gluconeogenesis and glycogenolysis—incretin-based medications are best able to reverse these adverse consequences and improve glycemia.

**Figure 4:**
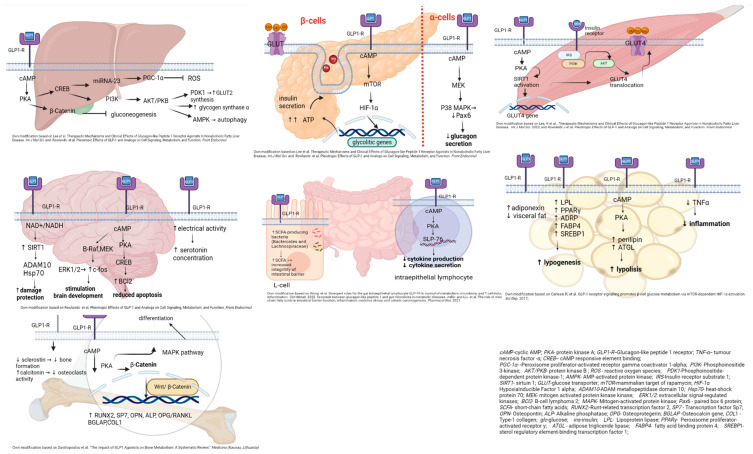
The effects of GLP-1A (GLP-1 agonists)/GLP1-RA activation on different tissues and organs affecting insulin resistance. cAMP: cyclic AMP; PKA: protein kinaseA; GLP1-R: Glucagon-like peptide 1 receptor; TNF-a: tumour necrosis factor-a; CREB-cAMP: responsive element binding; PGC-1a: Peroxisome proliferator-activated receptor gamma coactivator 1-alpha; PI3K: Phosphoinositide3-kinase; AKT/PKB: protein kinaseB; ROS: reactive oxygen species;PDK1: Phosphoinositide dependent protein kinase-1;AMPK-AMP: activated protein kinase; IRS: Insulin receptor substrate 1; SIRT1:sirtuin 1; GLUT: glucose transporter; mTOR: mammalian target of rapamycin; HIF-1a: Hypoxialnducible Factor 1 alpha; ADAM10: ADAM metallopeptidase domain 10; Hsp70: heat-shock protein 70; MEK: mitogen activated protein kinase kinase; ERK1/2: extracellular signal-regulated kinases; BCI2: B-cell lymphoma 2; MAPK: Mitogen-activated protein kinase; Pax6: paired box 6 protein; SCFA: short-chain fatty acids; RUNX2: Runt-related transcription factor 2; SP7: Transcription factor Sp7; OPN: Osteopontin; ALP: Alkaline phosphatase; OPG: Osteoprotegerin; BGLAP: Osteocalcin gene,COL1-Type-1 collagen; gloglucose; ins-insulin; LPL: Lipoprotein lipase; PPARy: Peroxisome proliferator- activated receptory; ATGL: adipose trigliceride lipase; FABP4: fatty acid binding protein 4; SREBP1: sterol regulatory element-binding transcription factor 1.

The effects of GLP-1A/GLP-1RA activation on different tissues are pictured in Figure 4, while Table 5 illustrates general mechanisms of reducing GS-induced insulin resistance and GS-induced hyperglycemia by means of GLP1-A.

### 6.5. Metformin

Despite the broad usage of metformin in diabetes treatment, its evidence of efficacy in SID or SIH is limited. Due to its slow onset of action and weak impact on postprandial glucose levels and glucose control during GS treatment, it is indicated for individuals treated with low doses of intermediate-acting GS for the long term [20].

### 6.6. Thiazolidinediones

The position of thiazolidinediones in the treatment of steroid-induced hyperglycemia is ambiguous. In people using low-dose glucocorticoid therapy treated with pioglitazone for 6 months, a statistically significant reduction in glucose concentration in OGTT and HbA1c was present [106]. Additionally, thiazolidinediones improve glucose control in severe GS-induced hyperglycemia when administered with insulin [107]. However, their side effects such as weight gain, fluid retention, and increased risk of bone fractures are analogous to those caused by glucocorticosteroids [108].

### 6.7. α-Glucosidase Inhibitors

α-glucosidase inhibitors are characterized by weaker anti-diabetic potency, immediate onset of action, and reduced postprandial plasma glucose level due to the inhibitory impact on the breakdown of complex carbohydrates and the blockage of the absorption of glucose in the digestive tract [109]. Their use in the treatment of SID/SIH is usually overlooked, as they are instead considered a further line of treatment. A beneficial effect on the reduction of postprandial hyperglycemia in combination with glinides has been described in patients with rheumatoid arthritis [87]. Table 6 contains a summary of the action profile of antidiabetic agents, including their use in the treatment of GS-induced hyperglycemia.

## 7. How to Choose the Proper Algorithm in SID/SIH Treatment

Some guidelines (such as those of the Polish Diabetes Society) recommend treatment of SID/SIH solely with insulin therapy. Considering the anabolic impact of insulin on glucose metabolism and the possibility of an increase in insulin resistance, it seems that not every patient should be treated with insulin therapy. No advantage of short-acting human insulins over insulin analogs in glycemic control has been demonstrated. Among oral drugs, the most commonly used are glinides and sulphonylurea derivatives. Metformin, despite its broad use in the treatment of type 2 diabetes, does not play an essential role in the treatment of SID. Rather, it is considered a supportive agent because it reduces insulin resistance. The use of thiazolidinediones in the first line of treatment is controversial. On the one hand, they reduce insulin resistance and improve glycemic control; but on the other, they deteriorate bone metabolism and induce heart failure. The share of GLP-1 analogs and DPP-4 inhibitors in the treatment of post-steroid glycemic disorders should increase, considering their beneficial effect on postprandial glycemia. Among the new antidiabetic drugs, flozins seem to be the least effective. Although no studies have been conducted on their effectiveness when used as monotherapy, in polytherapy they only slightly improve glycemic control without affecting HbA1c.

**Figure 5:**
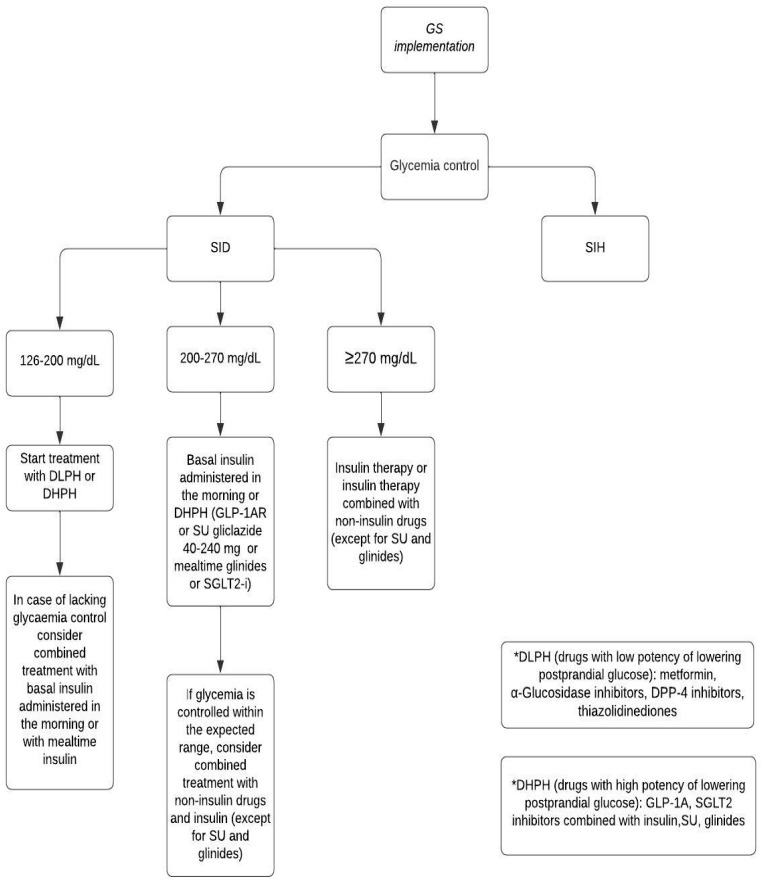
Proposed new algorithm of SID pharmacotherapy with the usage of the newest antidiabetic agents, own modification based on [26]. SID: steroid-induced diabetes; SIH: steroid-induced hyperglycemia; GLP-1RA: glucagon-like-peptide 1 receptor agonists; DLPH: drugs with low potency of lowering postprandial glucose; DHPH: drugs with high potency of lowering postprandial glucose; SU: sulphonylureas; SGLT2i: sodium-glucose cotransporter-2 inhibitors; DPP-4: inhibitors- dipeptidyl peptidase 4 inhibitors.

**Figure 6:**
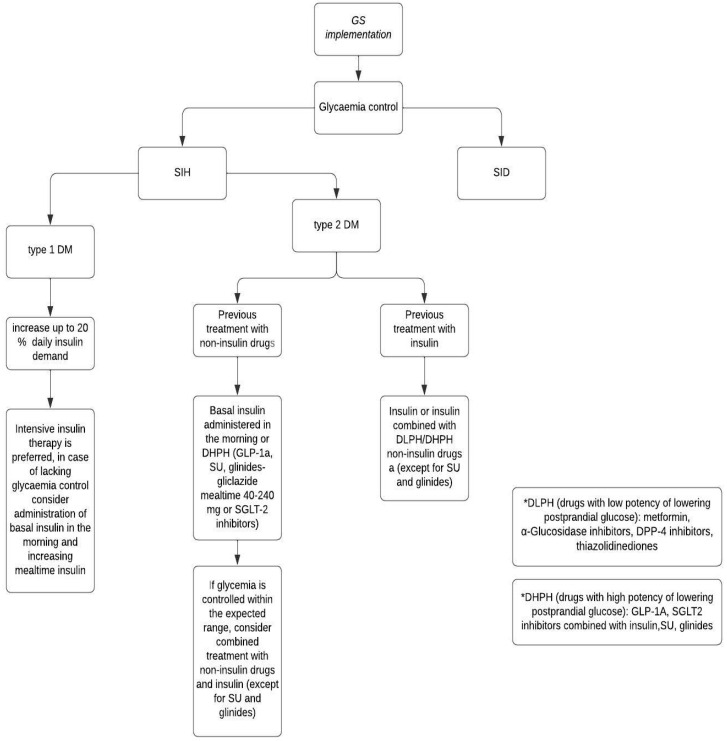
Proposed new algorithm of SIH pharmacotherapy with the usage of the newest antidiabetic agents, own modification based on [1]. DM: diabetes mellitus; SID: steroid-induced diabetes; SIH: steroid-induced hyperglycemia; GLP-1RA: glucagon-like-peptide 1 receptor agonists; DLPH: drugs with low potency of lowering postprandial glucose; DHPH: drugs with high potency of lowering postprandial glucose; SU: sulphonylureas; SGLT2i: sodium-glucose cotransporter-2 inhibitors; DPP-4: inhibitors- dipeptidyl peptidase 4 inhibitors.

When selecting a treatment regimen, the patient’s cardiovascular risk, the likelihood of bone fracture, and the financial capabilities of the patient should be considered. Some agents have positive cardiovascular action, which can reduce negative GS impact on the cardiovascular system. For example, metformin therapy reduces pro-inflammatory cytokine levels and improves endothelial function, leading to a decreased risk of atherosclerosis and improved vascular function [110,111]. GLP-1 agonists lower blood pressure by affecting the Na/H channel in the proximal tubule and protect against cardiac hypertrophy [94,112]. Additionally, they protect against the development of atherosclerosis and inhibit the progression of existing atherosclerotic plaques [113]. Data regarding the impact of DPP-4 inhibitors on the cardiovascular system are inconsistent. Most studies indicate their beneficial effects due to anti-inflammatory actions, improvement in endothelial function, and reduction in triglyceride, LDL-cholesterol, and non-HDL cholesterol levels [114,115,116]. Gliptins may also lower both systolic and diastolic blood pressure [117]. However, Nakamura et al. demonstrated that treatment with sitagliptin for 12 months had no beneficial effect on endothelial function [118]. Furthermore, saxagliptin and alogliptin may increase the risk of hospitalization due to heart failure [119]. SGLT2 inhibitors reduce the progression of atherosclerosis by decreasing oxidative stress, reversing endothelial dysfunction, and promoting vascular proliferation [120,121]. However, an increased risk of fracture during canagliflozin therapy due to decreased bone mineral density has been described [122]. The effect of pioglitazone on the cardiovascular system is unclear. It may induce endothelial progenitor cells, but may also promote the progression of existing atherosclerotic plaques [123,124]. Moreover, similarly to canagliflozin, thiazolidinediones may increase the risk of fracture [125,126]. Insulin secretagogues (sulfonylureas and meglitinides) are associated with an increased cardiovascular risk due to their inhibiting effect on ATP-sensitive potassium channels in the cardiovascular system [127]. In the U.K. Prospective Diabetes Study, no differences in cardiovascular outcomes were found among patients treated with insulin, glibenclamide, and chlorpropamide [128]. Alpha-glucosidase inhibitors can improve atherogenic dyslipidemia and oxidative stress by reducing postprandial glycemia and decreasing insulin resistance [129]. Figure 5 and Figure 6 present the algorithms of pharmacotherapy for SID/SIH including the newest anti-diabetic agents.

## 8. Limitations

Currently, there are no up-to-date studies comparing the effects of various preparations on carbohydrate metabolism disorders induced by glucocorticoid therapy. Considering the previously published results limited to a few studies and their mechanisms of action, including favorable effects on the cardiovascular system, incretin mimetics deserve particular attention in this indication [93,95,96,97,98,130,131,132]. Unfortunately, even with positive findings from animal model studies, it should be emphasized that this effect may differ in humans.

Clinicians are not always able to determine whether steroid-induced diabetes (SID) contains elements of steroid-induced hyperglycemia (SIH), as routine assessments of carbohydrate metabolism are not conducted, or due to the acute nature of symptoms that precludes delaying steroid administration. Most preparations are not registered for the treatment of post-steroid diabetes, so their use for this indication is considered off-label.

## 9. Conclusions

Despite the widespread introduction of biological drugs into the treatment of autoimmune and chronic inflammatory diseases, the use of glucocorticoids is still present in everyday clinical practice. The genomic mechanism of action of steroids involves long-lasting effects such as hyperglycemia which may persist for many weeks after their discontinuation. In many cases, the occurrence of diabetes is not diagnosed before the glucocorticoids are commenced and glucose levels are not monitored during the therapy. In the article, we present a new diagnostic and therapeutic algorithm for steroid-induced diabetes, taking into account various ranges of glycemia levels and the coexistence of type 1 and 2 diabetes in a person treated with glucocorticoids. As a rule, significant clinical symptoms of hyperglycemia prompt doctors to control glucose levels and consequently initiate treatment, often with insulin. On the one hand, insulin therapy limits the potential consequences of hyperglycemia including acute conditions such as acidosis or a hyperosmolar hyperglycemic state. On the other hand, when administered for a long time, it has an anabolic effect, inducing a vicious cycle of behavior from increased appetite, the subsequent increase in glucose levels, again increasing the need for insulin, and ultimately a constant increase in body weight. In the era of new anti-diabetic options, their early use should be considered to reduce the demand for insulin and limit insulin resistance induced by glucocorticoids.

Our study indicates the need to modify these recommendations, particularly regarding the use of incretin mimetics. Most societies recommend the use of sulfonylureas, meglitinides, and insulin therapy in the treatment of SID and SIH. Among the therapeutic proposals, GLP-1 agonists may be of particular importance in reducing postprandial hyperglycemia. We present the mechanism of action of GLP-1 analogs in counteracting the adverse effects of glucocorticoid therapy.

## Figures and Tables

**Table 1:** Risk factors that a physician should consider when treating a patient undergoing therapy with glucocorticoids that may lead to the earlier onset of diabetes.

Dose and time of GS treatment [3,20]
Age above 60 years [21,22] however, some studies indicate that age has no significant impact [19]
History of impaired fasting glucose or impaired glucose tolerance [23,24,25]
History of gestational diabetes [26], PCOS, or diabetes in familiar history [27]
BMI > 25 kg/m^2^ [21,22,28] but a meta-analysis of Deutsch et al. shows that BMI is not significantly associated with developing steroid-induced hyperglycemia.
Concentration of HBA1c > 6% [29]
Smoking [21]
Race (increased risk of SID in black individuals) [30]
History of cytomegalovirus (CMV) infection [31]
Specific HLA—type A30, B27, Bw42 [32]
Treatment with loop diuretics or impaired renal function (eGFR < 40 mL/min/1.73 m^2^) [27,29]
Genetic factors—glucocorticoid-receptor (GR) sensitivity for steroid hormone is genetically determined [4]. The polymorphism of N363S and Bcll results in increased GR sensitivity and leads to an increased risk of diabetes development, obesity, insulin resistance, and hypertension [33,34]. In contrast, polymorphisms ER22/E23K and A3669G lead to decreased GR sensitivity, which is associated with a lower risk of SID and SIH [35].

BMI: body mass index; PCOS: polycystic ovarian syndrome; HLA: human leukocyte antigens; SID: steroid-induced diabetes; SIH: steroid-induced hyperglycemia; GR: glucocorticoid receptor; CMV: cytomegalovirus.

**Table 2:** Pharmacological characteristics of most commonly used glucocorticoids based on [6,38].

Pharmaceutical Agent	Peak of Hyperglycaemic Effect (Hours)	Duration of Action	Approximate Equivalent Dose (mg)	Anti-Inflammatory Activity (Ratio) *
Hydrocortisone Prednisone Prednisolone	3	8–12 h	20	1.0
	8	12–36 h	5	3.5
	8	12–36 h	5	4.0
Methylprednisolone Dexamethasone	8	12–36 h	4	5.0
	Variable	36–54 h	0.75	30

* The ratio of anti-inflammatory effects is measured by the reference of hydrogenation as 1.

**Table 3:** Selected indications for the use of glucocorticosteroids, along with dosages.

Indication	Dose and Type of Steroid
Sudden hearing loss	Prednisone 1 mg/kg body weight per day (usually maximum dose 60 mg/day)/methylprednisolone 48 mg/day or dexamethasone 10 mg/day for 7–14 days [40]
Rheumatoid arthritis	Induction of remission–prednisolone 30 mg for 3 months Chronic doses in selected patients 2.5–10 mg prednisone [41]
Polymyalgia rheumatica	Prednisone 12.5–25 mg for 2–4 weeks [42]
Bell’s palsy	Prednisolone in a single dose of 60 mg for 5 days or 50 mg in two separate doses for 10 days [43]
Exacerbation of chronic obstructive pulmonary disease	Prednisone 40 mg for 5 days [44]
Prevention of vomiting after chemotherapy (high emetogenic potential)	Dexamethasone 12 mg 30 min before starting chemotherapy and dexamethasone 8 mg on days 2–4 [45]
Malignant spinal cord compression (MSCC)	Starting dose—dexamethasone 16 mg p.o. or i.v. then 16 mg once daily before noon is advised [46]
Croup syndrome	Dexamethasone once at a dose of 0.15–0.6 mg/kg body weight or prednisolone at a dose of 1 mg/kg body weight [43]
Graves orbitopathy	Methylprednisolone 500 mg intravenous every 7 days for 6 weeks, methylprednisolone 250 mg intravenous every 7 days for 6 weeks [47]

MSCC: malignant spinal cord compression.

**Table 4:** Proposed regimen of glycemia monitoring based on [5,26].

Monitoring of glycemia during GS treatment in hospitalized patient	Monitoring of glycemia during GS treatment in outpatient care
Previously recognized diabetes—at minimum 4 times a day, especially between 12 p.m.–4 p.m. and 2 h after meal	When starting GS treatment (after exclusion of DM before GS commencement), blood glucose level should be monitored at a minimum of 2–3 days after initiation therapy, especially between 12 p.m.–4 p.m. and 2 h after meal
No history of diabetes mellitus—blood glucose level measurement at least twice after starting GS treatment	In case of glycemia between 108–180 mg/dL, monitoring should be conducted 2 times a week. In case of glycemia ≥ 200 mg intensify glucose monitoring to 4 times a day
In case of blood glucose level ≥ 200 mg/dL in previously non-diabetic patients, intensify glucose monitoring to 4 times a day	In patients with implemented GS without previously assessed risk of diabetes (postprandial glycemia at least twice a day and HbA1c level should be measured)
	In the case of oncological patients treated with GS, glucose levels should be monitored at every visit

* Monitor Other Hemodynamic and Biochemical Parameters: BP, HR, Lipid Profile, creatinine, General Urine Test with Urinary Albumin-to-Creatinine ratio (UACaR); ** Before GS commencement, assess the fundus of the eye, then following ADA/PDS recommendations; *** After ending GS therapy in case of proper glycemia control, reassessment of OGTT and HbA1c levels should be performed after 3 and 6 months; DM: diabetes mellitus; GS: glucocorticoids; HbA1c: glycated hemoglobin.

**Table 5:** The mechanism of GLP-1A/GLP1-RA activation on insulin resistance and GS-induced hyperglycemia based on [91,92,101,102,103,104,105].

Organ/Tissue	The Influence of GLP-1A/GLP-1RA on Organ/Tissue	The Potential Influence of GLP1A/GLP1-RA on Reversal GS Impact on Organ/Tissue
Liver	Improve insulin sensitivity of hepatocytes ↓ de novo lipogenesis decreasing of liver fat content ↓ hepatic glucose production ↓ stellate cell activation ↓ steatosis ↓ NASH, MAFLD ↓ endoplasmic reticulum stress ↓ oxidative stress	↓ oxidative stress ↓ hepatic steatosis
Pancreatic insular system	↑ insulin secretion and biosynthesis ↑ β-cells proliferation ↓ β-cells apoptosis ↓ glucagone secretion↓ oxidative stress	Improve β-cells secretory function ↓ oxidative stress protection against β-cells apoptosis↑ β-cells proliferation
Skeletal muscles	↑ insulin sensitivity ↑ glucose uptake ↓ muscle inflammation improving muscle degeneration and muscle atrophy	↓ muscle insulin resistance Improves muscle degeneration and prevents muscle atrophy ↓ oxidative stress ↑ glucose uptake
Bones	↑ osteoblasts differentiation ↑ bone formation and ↑ osteocalcin level ↓ risk of fractures improve insulin sensitivity and glucose metabolism in bones	Improve bone metabolism ↓ the risk of osteoporosis and fractures Improve insulin sensitivity direct and indirect via weight loss
Brain	Loss of appetite ↑ POMC and ↑ CART ↓ NPY and ↓ AgRP Increase insulin sensitivity in the brain	Stimulate neuroprotection Improve cognitive functions Enhancing psychical states ↓ appetite, influencing positive on body weight
Intestines	Regulate gut motility Improve absorption of nutrients Improve intestinal barrier by up-regulation of gut microbiome ↓ inflammatory state Prevent oxidative stress	Through GLP-1 receptor present on intestinal epithelial cells, neuronal cells responsible for gut movement and immune system impact on reduction of gut barrier permeability for toxins and bacterias
White adipose Tissue	Enhancing insulin sensitivity ↓ lipolysis, ↓ lipogenesis, ↓ adipogenesis ↓ TNF-α; ↓ IL-6 ↓ leptin ↑ adiponectin ↓ stellate cell activation ↓ steatosis ↓ oxidative stress ↓ of visceral fat content	Improve insulin sensitivity Promote weight loss ↓ visceral fat

NASH: nonalcoholic steatohepatitis; MAFLD: metabolic dysfunction-associated fatty liver disease; GLP-1: glucagon-like peptide-1; GLP1-RA: glucagon-like peptide-1 receptor agonists; GLP-1A: glucagon-like peptide-1 agonists; TNF-α: tumor necrosis factor-α; IL-6: Interleukin 6; POMC-: proopiomelanocortin ;CART: cocaine- and amphetamine-regulated transcript; NPY: neuropeptide Y; AgRP: agouti-related peptide.

**Table 6:** Summary of the action profile of antidiabetic agents, including their use in the treatment of GS-induced hyperglycemia.

	GS Prednisolone	SU	Glinides	Metformin	Thiazolidinediones	GLP-1RA	DPP-4-Inhibitors	SGLT2i	α-Glucosidase
FPG	Low or sufficient ↑ during high doses	↓	↔ * mean glucose level	↓	Insufficient data	↓/Insufficient data * improve mean glucose level	↔/insufficient data * improve mean glucose level	↔/insufficient data	↓/↔
PPG	↑	↔/↓	↓↓	↓ improves AUC of glucose during OGTT	↔/Insufficient data Improves AUC of glucose during OGTT	↓↓	↓/↔	↔	↓ in combination with glinides
HbA1c	↑	↓↓↓	↓↓ * mean HbA1c	↓	↓	↓↓↓	↓	↓↓	↓
Weight	↑	↑/↔	↔	↔	↑	↓↓↓	↔	↓↓	↔/↓
Cautions and disadvantage	Resulting from side effects of GS	Risk of hypoglycemia	Frequent dosage, low availability	Caution should be exercised in patients with renal failure	Exacerbation of side effects GS in fluid retention	Gastrointestinal side effects, high cost	Gastrointestinal side effects,	Exacerbation of GS side effects in the field of bone fractures	Weak hypoglycemic effect
Cardiovascular risk	↑/↓	↔/↑	↔/↑	↓	↑	↓	↑/↔/↓	↔/↓	↔
Risk of fracture	↑	↔	↔	↓	↑	↑/↔/↓	↔/↓	↑	↔
Suitable for the type of GS	not applicable	Long-acting or intermediate-acting dosed 2 a day	Short-acting	Intermediate-acting	Intermediate-acting	Intermediate-acting or long-acting	Intermediate-acting or long-acting	Insufficient data (not tested in monotherapy)	Insufficient data (not tested in monotherapy)

AUC: area under the curve ; FPG: fasting plasma glucose; PPG: postprandial glucose; SU: sulphonylureas; SGLT2i: sodium glucose cotransporter-2 inhibitors; GS: glucocorticoids; OGTT: oral glucose tolerance test; GLP-1RA: glucagon like peptide 1 receptor agonists.
